# Tracking the evolution of non-headache symptoms through the migraine attack

**DOI:** 10.1186/s10194-022-01525-6

**Published:** 2022-11-23

**Authors:** Roberta Messina, Ilaria Cetta, Bruno Colombo, Massimo Filippi

**Affiliations:** 1 Neuroimaging Research Unit, Division of Neuroscience, IRCCS San Raffaele Scientific Institute, Via Olgettina, 60, 20132 Milan, Italy; 2 Neurology Unit, IRCCS San Raffaele Scientific Institute, Milan, Italy; 3 Vita-Salute San Raffaele University, Milan, Italy; 4 Neurorehabilitation Unit, IRCCS San Raffaele Scientific Institute, Milan, Italy; 5 Neurophysiology Service, IRCCS San Raffaele Scientific Institute, Milan, Italy

**Keywords:** Migraine, Non-headache symptoms, Disability, Clinical phenotype

## Abstract

**Background:**

The migraine attack is classically divided into the prodromal, aura, headache and postdromal phase. Previous studies have highlighted non-headache symptoms associated with migraine occurring during the prodromal or postdromal phase. This study aimed to track the evolution of non-headache symptoms throughout all phases of the migraine attack. We also wished to delineate the phenotype of patients with more symptomatic migraine episodes and explore the association between non-painful symptoms and migraine disease activity and patients’ disability.

**Methods:**

Two-hundred and twenty-five migraine patients were enrolled and were asked to recall retrospectively whether non-headache symptoms occurred during the prodromal, headache and postdromal phase of their attacks. The occurrence of symptoms during the different migraine phases was tested using the Cochran’s Q tests, Cohen’s and Fleiss’ kappa. Differences between groups according to the presence of non-headache symptoms through the entire migraine attack and correlations between the frequency of non-headache symptoms experienced during all phases and patients’ disease activity and disability were also assessed.

**Results:**

Ninety-nine percent of patients reported having at least one non-headache symptom in one phase of the migraine attack and 54% of patients had at least one non-headache symptom occurring during all phases of migraine. The occurrence of non-headache symptoms was different throughout the three phases of migraine, being higher during the headache phase than during the prodromal and postdromal phases. Symptoms with the highest co-occurrence throughout all migraine phases were neck stiffness, thirst and abdominal pain. Patients who experienced non-headache symptoms during all three phases of migraine were more frequently females, had a higher disability, were suffering from chronic migraine and had more frequently medication overuse headache.

**Conclusion:**

Migraine is a complex neurological disorder with a wide constellation of non-headache symptoms that can affect the burden of the disease. A better characterization of the evolution of non-headache symptoms through the different phases of migraine can enrich our knowledge on migraine pathophysiology and improve the management of the disease.

## Background

Migraine affects around 1 billion people worldwide and is one of the leading causes of disability in people younger than 50 years [1, 2]. Migraine is not simply a disorder of recurrent pain attacks but a complex and multifaceted neurological disease. Although head pain is the main symptom characterizing the migraine attack, migraine patients can also experience a plethora of non-headache symptoms starting before pain onset or persisting after headache resolution [3]. The migraine attack has been classically divided into the prodromal, aura, headache and postdromal phases. Head pain, by its presence or absence, marks the passage from one phase to another one [4]. Before the headache onset, numerous patients may experience premonitory symptoms, including mood changes, irritability, difficulty in concentration, food cravings, repetitive yawning, increased sensitivity to external stimuli, cranial autonomic symptoms and neck stiffness [5, 6]. During the headache phase, the migraine pain can be accompanied by nausea, vomiting, phonophobia and photophobia [2]. A variety of symptoms, including fatigue, sensory hypersensitivity, concentration difficulties, mood changes and neck stiffness, may persist for up to 24 h after headache pain resolution, thus characterizing the postdromal phase [7, 8]. Although migraine phases are usually described as consecutive stages, some non-headache symptoms may start during the earliest phase of the migraine attack and continue until the postdromal phase [9]. Previous studies investigated the prevalence and phenotype of non-headache symptoms occurring during the prodromal or postdromal phase [5, 6, 8, 10]. Only a few studies [5, 10] have explored the occurrence of the same non-headache symptom throughout all phases of the migraine attack. Recent magnetic resonance imaging studies showed that activation of brainstem, diencephalic and cortical areas could mediate non-painful symptoms associated with migraine headache [11–14]. Understanding the evolution of non-headache symptoms through the different phases of migraine could enrich our knowledge on migraine pathophysiology and improve the management of the disease.

The aim of this study was to characterize non-headache symptoms in a large sample of migraine patients and track the evolution of such symptomatology through the different phases of the migraine attack. We also sought to delineate the phenotype of patients experiencing non-headache symptoms during all phases of migraine and explore the association between non-painful symptoms and migraine disease activity and patients’ disability.

## Methods

We enrolled 225 patients with migraine who have attended the Headache Clinic at San Raffaele Hospital for their first clinic appointment from February 2019 to July 2021. Inclusion criteria were: age ≥ 18 years; a diagnosis of migraine based on the International Classification of Headache Disorder criteria (third edition) [15]; evidence of a personally signed and dated informed consent document indicating that the subject had been informed of all pertinent aspects of the study. Exclusion criteria of the study included other primary headache disorders, other chronic pain conditions and the inability to provide a detailed clinical history. Patients taking migraine preventive treatments, with both episodic and chronic migraine, with a history of medication overuse and with comorbid depression or anxiety were included in the study. All patients underwent a neurological examination and the following clinical features of migraine were collected: age of migraine onset, frequency of monthly headache days (MHD), frequency of monthly migraine days (MMD), ongoing preventive treatments, migraine prevention used in the past and abortive treatments currently used to stop migraine attacks. The severity of attacks was determined as regards the severity of pain according the Numerical Rating Scale (NRS) [16] and presence of cutaneous allodynia during the migraine attack, quantified using the Allodynia Symptom Checklist (ASC-12) [17]. Patients’ disability was also quantified using the Migraine Disability Assessment Test (MIDAS) [18] and Headache Impact Test (HIT-6) [19]. During a semi-structured clinical interview, non-headache symptoms were orally listed and patients were asked to recall retrospectively whether each symptom usually occurred during their migraine attacks and whether these symptoms occurred during the prodromal, headache and postdromal phase of migraine. It was specified to patients to report the occurrence of non-headache symptoms taking into account those periods when they were not taking any migraine prevention and regardless the use of acute treatments. Non-headache symptoms investigated in migraine patients during the clinical interview are reported in Table 1.

**Table 1:** List of non-headache symptoms investigated in migraine patients

Category	Symptoms
Fatigue/Cognitive changes	Irritability
	Difficulty in concentration
	Fatigue
	Lethargy
	Mood disturbance
	Speech difficulty
Homeostatic alterations	Yawning
	Food craving
	Food aversion
	Thirst
	Frequent urination
Sensory sensitivities	Photophobia
	Phonophobia
	Neck stiffness
Gastrointestinal symptoms	Nausea
	Abdominal pain

### Standard protocol approvals

The study was approved by the local ethical committee and written informed consent was obtained from all participants.

### Statistical analysis

Based on previous studies [20, 21], we estimated that a sample of at least 100 patients would allow solid results. The Cochran’s Q test was performed to assess any significant differences in the frequency of non-headache symptoms across the three phases of the migraine attack. The co-occurrence of symptoms during the different phases of migraine was tested using Cohen’s and Fleiss’ kappa coefficients with 95% confidence intervals. For the Cohen’s and Fleiss’ kappa coefficients we considered significant a kappa value > 0.3 (fair-good agreement), with a *p* <  0.05. Patients were divided into two subgroups according to the presence of non-headache symptoms through the entire migraine attack. Between-group differences in demographic and clinical characteristics were assessed using the independent *t* test or Mann-Whitney test for continuous variables and the Fisher exact test for categorical variables (version 26.0; SPSS software, IBM, Armonk, NY). Correlations between the frequency of non-headache symptoms experienced during all phases of migraine and patients’ disease activity and disability were assessed using the Spearman’s correlation test.

## Results

The main demographic and clinical characteristics of migraine patients are summarized in Table 2. The mean age of subjects was 45 years (range: 19–76), 174 (77%) were women and 48 (21%) patients suffered from migraine with aura. One hundred and twenty-five (56%) patients had chronic migraine and medication overuse was present in 112 (49%) cases. Eighty-one (36%) patients were taking preventive treatments including beta-blockers, antiepileptics, tricyclic antidepressants and Onabotulinumtoxin A. Enrolled patients could have failed preventives in the past with a median of three treatments. Seventeen (7%) patients suffered from depression and 7 (3%) patients had a history of anxiety. On average, patients reported having migraine attacks with a moderate pain intensity and mild cutaneous allodynia. Patients were severely disabled by migraine and reported a severe impact of migraine on their life (Table 2).

**Table 2:** Main demographic and clinical characteristics of migraine patients enrolled in the study

Median age [IQ range] (years)	46 [34–54]
Women/Men	174 (77%) - 51 (23%)
Median disease duration [IQ range] (years)	30 [18–39]
Migraine with aura/Migraine without aura	48 (21%) – 177 (79%)
Median monthly headache days [IQ range]	15 [8–26]
Median monthly migraine days [IQ range]	12 [8–18]
Episodic/Chronic migraine	100 (44%) – 125 (56%)
Median of preventive treatments used in the past [IQ range]	3 [0.5–5]
Migraine with medication overuse/ Migraine without medication overuse	112 (49%) – 113 (51%)
Median of monthly tablets of acute treatments [IQ range]	15 [8–26]
Median MIDAS score [IQ range]	36 [15–89]
Median HIT-6 score [IQ range]	65 [61–68]
Median NRS score [IQ range]	8 [7–8.5]
Median ASC-12 score [IQ range]	4 [1–7]

Measures are reported as median and interquartile range (25th – 75th percentiles). Sex, type of migraine, presence of aura and medication overuse are reported as frequencies

Abbreviations: *ASC-12* Allodynia Symptom Checklist, *HIT-6* Headache Impact Test, *IQ* interquartile range, *MIDAS* Migraine Disability Assessment Test, *NRS* Numeric Rating Scale (NRS)

### Non-headache symptoms during the prodromal, headache and postdromal phase

Two hundred twenty-three (99%) patients reported having at least one non-headache symptom in one phase of their migraine attacks. One hundred and ninety five (87%) patients reported experiencing at least one non-headache symptom in two phases of their attacks and 122 (54%) patients had at least one non-headache symptom throughout all three phases of migraine. The mean number of non-headache symptoms occurring throughout the entire migraine attack was 1.24 (range: 0–10).

**Fig. 1:**
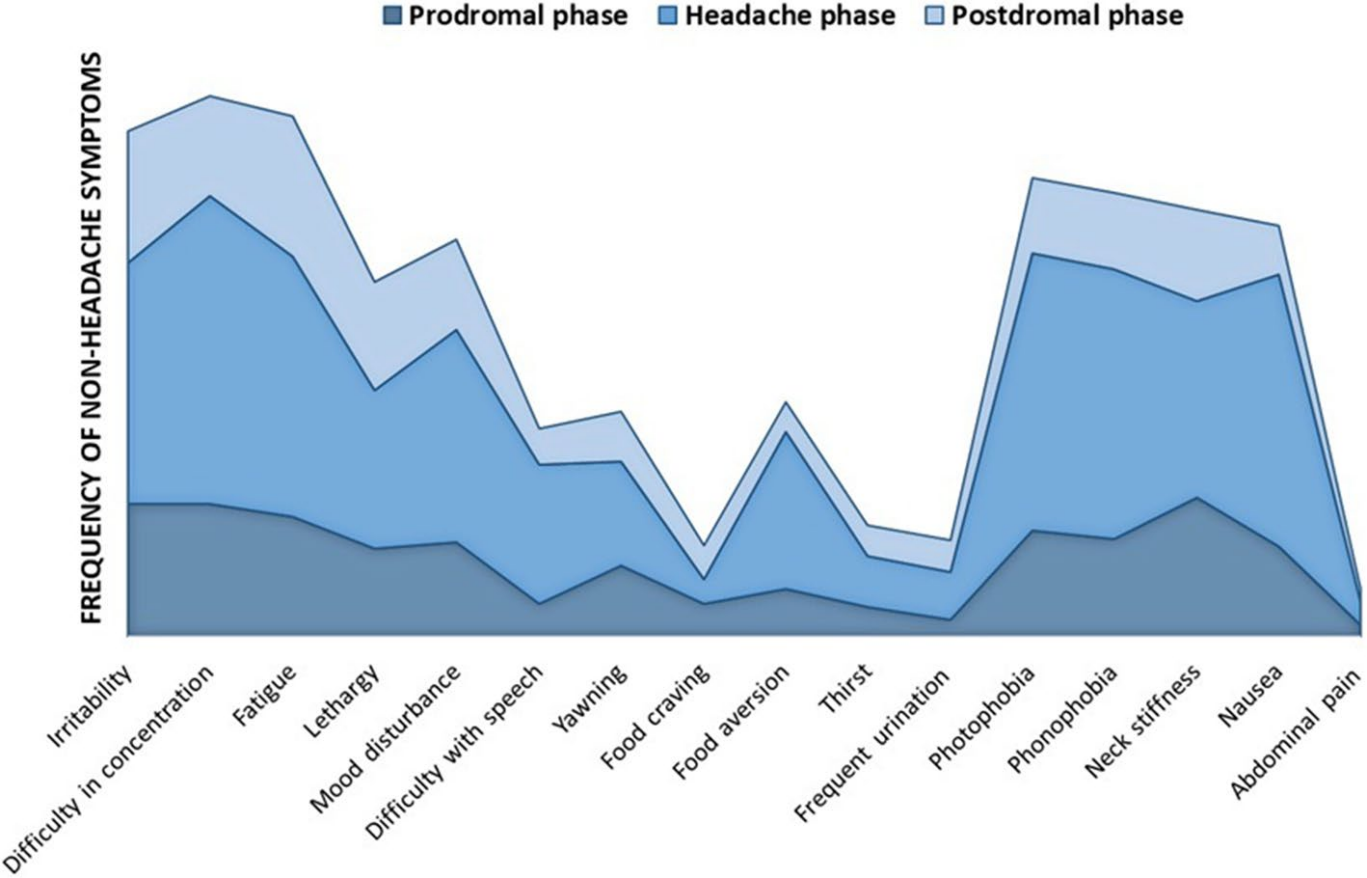
Occurrence of non-headache symptoms during the different phases of migraine. Frequencies of non-headache symptoms occurring during the prodromal (dark blue), headache (blue) and postdromal (light blue) phase

Apart from food craving, the occurrence of non-headache symptoms was different throughout the three phases of migraine (Cochran’s Q test: *p* <  0.001), being higher during the headache phase than during the prodromal and postdromal phase. The occurrence of food craving did not significantly change over the migraine attack (Cochran’s Q test: *p* = 0.341) (Fig. 1). The most common reported non-headache symptom in the prodromal phase was neck stiffness (*n* = 88, 39% of patients), while difficulty in concentration was most frequently reported during the headache phase (*n* = 196, 87%) and fatigue (*n* = 89, 40%) during the postdromal phase (Fig. 1). Symptoms with the highest co-occurrence through all three phases were neck stiffness, thirst and abdominal pain (Table 3). No significant differences in the occurrence of non-headache symptoms were found between patients with and without aura, patients on migraine prevention and patients who were not taking preventive treatments as well as between episodic and chronic patients.

**Table 3:** Co-occurrence of non-headache symptoms during all three phases of migraine

Non-headache symptoms	k value	*p* value^*^	C.I. 95%
Nausea	0.007	0.861	0.004–0.009
Difficulty in concentration	0.025	0.508	0.028–0.023
Photophobia	0.034	0.384	0.031–0.036
Speech difficulty	0.038	< 0.001	0.197–0.202
Fatigue	0.051	0.182	0.049–0.054
Irritability	0.060	0.118	0.058–0.063
Phonophobia	0.086	0.025	0.084–0.089
Food aversion	0.113	0.003	0.110–0.115
Frequent urination	0.135	< 0.001	0.133–0.137
Mood disturbance	0.215	< 0.001	0.212–0.217
Yawning	0.228	< 0.001	0.226–0.230
Food craving	0.246	< 0.001	0.243–0.248
Lethargy	0.247	< 0.001	0.244–0.249
Thirst	0.323	< 0.001	0.321–0.326
Neck stiffness	0.396	< 0.001	0.393–0.398
Abdominal pain	0.581	< 0.001	0.579–0.584

Abbreviations: *CI* Confidence interval

^*^Fleiss’ kappa coefficients with 95% CIs. Kappa value > 0.3 (fair-good agreement) with a *p* <  0.05 was considered significant

Pairing each phase of migraine in couple of two, we found that if lethargy, thirst and food craving occur during the prodromal phase, it is high likely that they will persist during the headache phase, while if phonophobia and mood disturbances occur during the prodromal phase, they will probably occur also during the postdromal phase. If patients report thirst during the headache phase, it is probable that they will experience such symptomatology also during the postdromal phase. The abdominal pain and neck stiffness if present tend to occur during all the three phases of migraine (Table 4).

**Table 4:** Co-occurrence of non-headache symptoms during paired migraine phases

Subject-reported symptoms	Migraine phases	Percentage agreement (%)	k value	***p*** value^*^	C.I. 95%
Irritability	Prodrome - Headache	52	0.128	0.020	0.024–0.0231
	Prodrome - Postdrome	60	0.162	0.005	0.037–0.280
	Headache - Postdrome	46	0.113	0.005	0.042–0.183
Difficulty in concentration	Prodrome - Headache	45	0.072	0.047	0.005–0.138
	Prodrome - Postdrome	64	0.198	0.004	0.060–0.320
	Headache - Postdrome	37	0.055	0.068	0.004–0.105
Fatigue	Prodrome - Headache	49	0.112	0.021	0.021–0.202
	Prodrome - Postdrome	57	0.075	0.256	0.056–0.206
	Headache - Postdrome	52	0.125	0.017	0.025–0.224
Lethargy	Prodrome - Headache	71	0.397	< 0.001	0.280–0.500
	Headache - Postdrome	60	0.173	0.007	0.048–0.298
	Prodrome - Postdrome	68	0.195	0.003	0.060–0.330
Yawning	Prodrome – Headache	73	0.297	< 0.001	0.142–0.416
	Headache - Postdrome	72	0.192	0.001	0.061–0.323
	Prodrome - Postdrome	78	0.237	< 0.001	0.084–0.389
Neck stiffness	Prodrome - Headache	75	0.505	< 0.001	0.400–0.610
	Headache - Postdrome	64	0.317	< 0.001	0.416–0.218
	Prodrome - Postdrome	74	0.424	< 0.001	0.305–0.543
Food aversion	Prodrome - Headache	63	0.206	< 0.001	0.108–0.304
	Headache - Postdrome	61	0.148	< 0.001	0.064–0.232
	Prodrome - Postdrome	83	0.058	< 0.001	0.110–0.226
Food craving	Prodrome - Headache	91	0.398	< 0.001	0.187–0.609
	Headache - Postdrome	88	0.207	0.001	0.009–0.405
	Prodrome - Postdrome	85	0.152	0.023	0.026–0.330
Frequent urination	Prodrome - Headache	88	0.291	< 0.001	0.103–0.479
	Headache - Postdrome	81	0.060	0.358	0.087–0.207
	Prodrome - Postdrome	88	0.070	0.267	0.096–0.236
Mood disturbance	Prodrome - Headache	61	0.293	< 0.001	0.199–0.387
	Headache - Postdrome	52	0.139	0.008	0.042–0.235
	Prodrome – Postdrome	75	0.365	< 0.001	0.228–0.502
Phonophobia	Prodrome - Headache	48	0.157	< 0.001	0.088–0.225
	Headache - Postdrome	41	0.093	0.011	0.032–0.153
	Prodrome - Postdrome	64	0.393	< 0.001	0.258–0.528
Photophobia	Prodrome - Headache	47	0.139	0.001	0.068–0.209
	Headache - Postdrome	39	0.073	0.032	0.016–0.129
	Prodrome - Postdrome	72	0.271	< 0.001	0.134–0.408
Thirst	Prodrome - Headache	88	0.408	< 0.001	0.228–0.588
	Headache - Postdrome	86	0.352	< 0.001	0.172–0.532
	Prodrome - Postdrome	87	0.186	0.005	0.006–0.378
Speech difficulty	Prodrome - Headache	69	0.255	< 0.001	0.156–0.354
	Headache - Postdrome	68	0.237	< 0.001	0.135–0.339
	Prodrome - Postdrome	87	0.244	< 0.001	0.053–0.434
Nausea	Prodrome - Headache	47	0.171	< 0.001	0.140–0.230
	Headache - Postdrome	36	0.080	0.005	0.069–0.229
	Prodrome - Postdrome	75	0.225	< 0.001	0.366–0.084
Abdominal pain	Prodrome - Headache	95	0.525	< 0.001	0.271–0.779
	Headache - Postdrome	95	0.498	< 0.001	0.752–0.244
	Prodrome - Postdrome	98	0.793	< 0.001	0.566–1.020

Abbreviations: *CI* Confidence interval

^*^Cohen’s kappa coefficients with 95% CIs. Kappa value > 0.3 (fair-good agreement) with a *p* < 0.05 was considered significant

### Phenotype of patients experiencing non-headache symptoms throughout the entire migraine attack

Sex, type of migraine and presence of medication overuse were significantly different between patients experiencing non-headache symptoms throughout all phases of migraine and those patients having non-headache symptoms in one or two phases of migraine, being the female gender, chronic migraine and presence of medication overuse more prevalent in patients with a higher number of migraine-associated symptoms. Compared to patients with less symptomatic migraine, patients having non-headache symptoms throughout all phases of migraine showed higher headache attack frequency, intake of acute migraine treatments, HIT-6 and MIDAS scores (Table 5).

**Table 5:** Phenotype of patients according the presence of non-headache symptoms throughout all three phases of migraine

	Patients having at least one non-headache symptom throughout all three phases of migraine	Patients having at least one non-headache symptom in one or two phases of migraine	*p* value^*^ Non-headache symptoms in all phases vs non-headache symptoms in one or two phases of migraine
**N° of subjects**	122 (54%)	103 (46%)	–
**Median age [IQ range] [years]**	45 [34–51]	47 [34–57]	0.099
**Women/Men**	104 (85%) – 18 (15%)	70 (68%) – 33 (32%)	0.002
**Median disease duration [IQ range]**	30 [16–39]	30 [20–40]	0.914
**Migraine with aura/Migraine without aura**	30 (25%) – 92 (75%)	18 (17%) – 85 (83%)	0.253
**Median monthly headache days [IQ range]**	20 [9–30]	12 [7–23]	0.001
**Median monthly migraine days [IQ range]**	15 [8–20]	11 [6–17]	0.056
**Episodic/Chronic migraine**	40 (33%) - 82 (67%)	57 (55%) – 46 (45%)	0.001
**Patients taking/not taking migraine prevention**	47 (39%)/ 75 (61%)	34 (33%)/ 69 (67%)	0.4
**Median of preventive treatments used**	3 [1–5]	3 [0–5]	0.153
**Migraine with medication overuse/ Migraine without medication overuse**	70 (57%) – 52 (43%)	40 (39%) – 63 (61%)	0.004
**Median of monthly tablets of acute treatments [IQ range]**	16 [8–30]	12 [5–20]	0.015
**Median MIDAS score [IQ range]**	55 [20–103]	34 [17–69]	0.004
**Median HIT-6 score [IQ range]**	66 [63–69]	64 [61–67]	0.002
**Median NRS score [IQ range]**	8 [7–9]	8 [7–9]	0.156
**Median ASC score [IQ range]**	5 [2–8]	4 [1–7]	0.130

^*^Independent *t* test or Mann-Whitney test for continuous variables and the Fisher exact test for categorical variables

### Correlations between non-headache symptoms and migraine disease activity and patients’ disability

The higher the number of non-headache symptoms experienced through all phases of migraine, the higher the MHD (*r* = 0.22, *p* <  0.001), MMD (*r* = 0.12, *p* = 0.02), intake of acute treatments (*r* = 0.16, *p* = 0.007), MIDAS (*r* = 0.19, *p* = 0.001), HIT-6 (*r* = 0.20, *p* = 0.001) and NRS (*r* = 0.09, *p* = 0.002) scores were.

## Discussion

In this study, we collected data about the occurrence of non-headache symptoms before, during and after the headache phase of the migraine attack in a large sample of migraine patients. In recent years, we have witnessed an increasing recognition of non-headache symptoms in association with migraine pain and higher interest in understanding their pathophysiology. Here, we showed that almost all patients experienced at least one non-headache symptom in one phase of the migraine attack and more than half of our patients experienced at least one non-headache symptom throughout all phases of migraine, suggesting that such symptoms are important migrainous features that need to be investigated.

Previous studies demonstrated that non-headache symptomatology may precede the headache phase by up to 72 hours and may persist for 1 day after the headache resolution in the postdromal phase [6, 10]. In line with previous studies, we found that the most common reported non-headache symptom in the prodromal phase was neck stiffness while fatigue was mostly reported during the postdromal phase [5, 8].

Although cognitive, mood and homeostatic changes are usually described as manifestations of the prodrome and postdrome phase [5, 6, 8, 10], our data showed that, when interrogated, migraine patients most often recall having non-headache symptoms in association to the headache pain. During the headache phase, beside the canonical migraine-specific symptoms such as nausea, photophobia and phonophobia, we found that difficulty in concentration was the most frequently reported symptom. This result is corroborated by previous magnetic resonance imaging studies showing an altered functional activity of brain networks involved in cognition in migraine patents during the headache phase of spontaneous and induced migraine attacks [22, 23].

Another interesting finding was that non-headache symptoms could start during any phase of the migraine attack and be present during all phases. These findings suggest that the notion of migraine phases coming in a linear and discrete succession may be too simplistic to explain the migraine attack. While migraine should be considered as a continuum with phases occurring in a sequential but overlapping manner.

Interestingly, patients with a higher number of migraine-associated symptoms were females, had a higher attack frequency, disability, intake of acute medications and a higher risk of medication overuse. These findings are consistent with previous data showing that female migraine patients experience a larger number of migraine symptoms, are more disabled by the disease and use greater healthcare resources compared to male patients [24, 25]. Hormonal fluctuations, different biology, genetic features and social environment may explain sex differences in the migraine phenotype [25]. Patients with more non-headache symptoms are those with a higher attack frequency and who require a higher intake of acute treatments, thus increasing the risk of medication overuse.

Our findings suggest that non-headache symptoms may play a role in determining the impact of migraine and patients’ disability. This speculation has important clinical, social and economic implications since patients with frequent symptomatic migraine attacks can have a poor quality of life and experience a large functional impact on their work, school and family life. Future studies assessing the migraine burden should therefore also take into account the repercussion of non-headache symptoms.

Our results provide further insights into the pathophysiology of the migraine attack. We found that neck stiffness, thirst, abdominal pain and food craving can occur throughout the prodromal, headache and postdromal phases of migraine. Of note, these symptoms can be mediated by the hypothalamus, via the orexinergic and dopaminergic systems [3, 4, 11, 12] suggesting its constant involvement during the migraine attack. In accordance with our findings, previous imaging studies have shown an early activation of the hypothalamic, trigeminal, pontine and thalamo-cortical pathways during the prodromal phase, which can persist during the headache phase [11, 22, 26, 27]. An early and persistent activation of the hypothalamic dopaminergic neurons may also explain the co-occurrence of lethargy during the prodromal and headache phase we have found in our sample of patients [11]. Pairing migraine phases in couple of two, we demonstrated that if phonophobia and mood disturbances occur during the prodromal phase, it is high likely that they will be present also during the postdromal phase. Based on these findings, it could be hypothesized an abnormal activity of the auditory, limbic and thalamic systems, which are respectively involved in sound and emotional processing, during the early and late stages of the attack.

The potential effect of acute and preventive treatments on non-headache symptoms has been poorly investigated. Only one study evaluated whether acute migraine treatments could ameliorate non-headache symptoms, showing no effects of triptans on such symptomatology during the postdromal phase [8]. Another recent study showed that patients treated with monoclonal antibodies targeting the calcitonin gene-related peptide (CGRP) pathway reported improvement of non-headache symptoms, like irritability and mood changes. Moreover, this study showed that anti-CGRP monoclonal antibodies could prevent not only the pain but also the onset of non-headache symptoms [28].

Our study is not without limitations. First, patients retrospectively recalled having non-headache symptoms during the different phases of migraine. When asked retrospectively, patients may fail to remember if non-headache symptoms occur during their attacks, especially chronic migraine patients who may lose the original migraine features of their attacks when evolving in a chronic form. Second, we cannot exclude that patients could have misinterpreted postdromal symptoms with acute treatments side effects, thus under- or overestimating the occurrence of postdromal symptomatology. In addition, differences between treated and untreated attacks were not evaluated. Third, the presence of comorbid psychiatric disorders in 24 patients may have influenced patients’ reporting of mood disturbances during the different phases of migraine. Fourth, the sample size of subgroups of patients (e.g. patients with and without aura) was relatively small. Future studies using headache diaries to prospectively collect data regarding the occurrence of non-headache symptoms are needed. Moreover the occurrence of non-headache symptoms during the aura phase should be explored in the future.

## Conclusions

Overall, our results confirm the notion that migraine is not simply characterized by recurring pain episodes but is a far more complex neurological disorder with a wide constellation of non-headache symptoms that can play a role in increasing the burden of the disease. Although the identification of distinct migraine phases may ease the study of their underlying mechanisms, it should be kept in mind that most non-headache symptoms may occur throughout all phases. A better characterization of the evolution of non-headache symptoms through the different phases of migraine can improve our understanding of its pathophysiology. Non-headache symptoms should be investigated in routine clinical practice and the effect of acute and preventive treatments on such symptomatology should be further explored in future studies.

## Data Availability

Data supporting the results of this study are available from the corresponding author, upon reasonable request.

## Abbreviations

ASC-12: Allodynia Symptom Checklist
CGRP: calcitonin gene-related peptide
HIT-6: Headache Impact Test
MIDAS: Migraine Disability Assessment Test
MHD: Monthly Headache Days
MMD: Monthly Migraine Days
NRS: Numerical Rating Scale
